# Limitations of A Measurement Tool to Assess Systematic Reviews (AMSTAR) and suggestions for improvement

**DOI:** 10.1186/s13643-016-0237-1

**Published:** 2016-04-12

**Authors:** Brittany U. Burda, Haley K. Holmer, Susan L. Norris

**Affiliations:** Kaiser Permanente Center for Health Research, 3800 N. Interstate Ave, Portland, OR 97227 USA; Oregon Health and Science University, 3181 SW Sam Jackson Park Road, Portland, OR 97239 USA; World Health Organization, Av. Appia 20, CH-1211, Geneva, 27 Switzerland

**Keywords:** Systematic review, Quality assessment, Risk of bias

## Abstract

A Measurement Tool to Assess Systematic Reviews (AMSTAR) is a commonly used tool to assess the quality of systematic reviews; however, modifications are needed to improve its usability, reliability, and validity. In this commentary, we summarize our experience and the experiences of others who have used AMSTAR and provide suggestions for its improvement. We propose that AMSTAR should modify a number of individual items and their instructions and responses to make them more congruent with an assessment of the methodologic quality of systematic reviews. We recommend adding new items and modifying existing items to assess the quality of the body of evidence and to address subgroup and sensitivity analyses. More detailed instructions are needed for scoring individual items across multiple reviewers, and we recommend that a total score should not be calculated. These suggestions need to be empirically tested prior to implementation.

## Background

A Measurement Tool to Assess Systematic Reviews (AMSTAR) is a commonly used tool to assess the methodologic quality of systematic reviews [1]. It has demonstrated satisfactory reliability and construct validity [2] for systematic reviews of randomized controlled trials of treatment interventions [3]. AMSTAR is widely used to assess the quality of systematic reviews, and some users state it is the most appropriate (and best) tool [4–6], while others have found it problematic [7–17] and therefore modified the tool [7, 11, 15, 18–30]. In this commentary, we summarize our experience using AMSTAR along with the experiences of others, describe several key issues, and provide suggestions for improvement (Table 1).

**Table 1:** Concerns regarding AMSTAR items, instructions, responses, and suggested revisions

AMSTAR tool^a^		Issues			Suggested revisions		
Item	Instructions	Related to the item	Related to the instructions	Related to the responses	Item	Instructions	Responses
1. Was an “a priori” design provided?	The research question and inclusion criteria should be established before the conduct of the review. Note: Need to refer to protocol, ethics approval, or pre-determined/a priori published research objectives to score “yes.”	The phrase “a priori design” is unclear.	Unless a protocol is available or the authors explicitly state that the design was developed a priori*,* a “yes” response is not indicated; thus “cannot answer” is likely the most common response. Many review authors state that they developed the research questions and inclusion criteria prior to executing the search; however, according to the instructions, a report of such an approach would still be “cannot answer” as there is no reference to a protocol, for example.	“Not applicable” is not an appropriate response.	Reword: Were the review questions and inclusion/exclusion criteria clearly delineated prior to executing the search strategy?	Reword: The review questions and inclusion/exclusion criteria should be established a priori as evidenced by a published protocol or an explicit statement in the review. Note: If the review refers to a protocol, ethics approval, or to pre-determined research questions and inclusion/exclusions criteria, score “yes.”	Remove the “not applicable” response.
2. Was there duplicate study selection and data extraction?	There should be at least two independent data extractors and a consensus procedure for disagreements should be in place. Note: Two people do study selection, two people do data extraction, consensus process or one person checks the other’s work.	None.	The main sentence relates to extraction only, and the “note” relates to the other aspects of the question. The “note” is not clearly written; for example, does the consensus process apply to study selection as well?	“Not applicable” is not an appropriate response.	None.	Reword: There should be at least two independent assessors for study selection (i.e., title, abstract and full-text screening). There should be either duplicate independent data extraction or verification of extracted data by a second person. A consensus process should be used when disagreements arise in either study selection at the full-text stage or in data extraction. Note: If two independent people do study selection and data extraction is verified, with consensus used in the event of disagreements, then indicate “yes.”	Remove the “not applicable” response.
3. Was a comprehensive literature search performed?	At least two electronic sources should be searched. The report must include years and databases used (e.g., Central, EMBASE, and MEDLINE). Key words and/or MESH terms must be stated and where feasible the search strategy should be provided. All searches should be supplemented by consulting current contents, reviews, textbooks, specialized registers, or experts in the particular field of study, and by reviewing the references in the studies found. Note: At least two sources plus one supplementary strategy used, select “yes.”	This item should proceed to the current item 2.	Additional clarity is needed and inclusion and exclusion criteria related to language of publication should be explicitly addressed.	“Not applicable” is not an appropriate response.	Reorder: This item should precede current item 2.	Reword: At least two bibliographic databases should be searched. The report must include years and databases examined (e.g., Central, EMBASE, and MEDLINE). Key words and/or MESH terms must be reported and the search strategy available. All searches should be supplemented by consulting reviews, specialized registers, or experts in the particular field of study, and by reviewing the references in the studies found. Publications in all relevant languages should be sought and a justification provided when there are language restrictions. Note: If at least two bibliographic databases plus one supplementary strategy were used, select “yes.”	Remove the “not applicable” response.
4. Was the status of publication (i.e. gray literature) used as an inclusion criterion?	The authors should state that they searched for reports regardless of their publication type. The authors should state whether or not they excluded any reports (from the systematic review), based on their publication status, language, etc. Note: If review indicates that there was a search for “gray literature” or “unpublished literature,” indicate “yes.” Single database, dissertations, conference proceedings, and trial registries are all considered gray for this purpose. If searching a source that contains both gray and non-gray, must specify that they were searching for gray/unpublished literature.	As written, this item is a reporting issue and not a quality issue. The item implies that if publication status was an inclusion (or exclusion) criterion, you respond “yes.” This differs from the instructions which focus on the appropriate inclusion of gray literature.	The second sentence suggests that the review simply has to state if any reports were excluded based on publication type, which is a reporting issue and not a quality issue. Language of publication is primarily an issue of gray literature.	“Not applicable” is not an appropriate response.	Reword: Was relevant gray literature included in the review? Reorder: This item should follow current item 3.	Reword: The authors searched for and considered gray literature (e.g., trial registries, conference abstracts, dissertations, and unpublished reports) as appropriate to the research question. Note: If the review indicates that there was a search for gray literature that is appropriate to the research question, score “yes.”	Remove the “not applicable” response.
5. Was a list of studies (included and excluded) provided?	A list of included and excluded studies should be provided. Note: Acceptable if the excluded studies are referenced. If there is an electronic link to the list, but the link is dead, select “no.”	None.	Including a list of all excluded studies may not be feasible, even if online capabilities are available. It is unclear at what stage the excluded list is focused; the full-text or the title and abstract stage.	“Not applicable” is not an appropriate response.	None.	Reword: A list of included and excluded studies at the full-text stage should be available to the reader (either within the publication, in an online appendix, or from the review authors). Note: If a list of both included and excluded studies (the latter at the full-text stage) is available either directly or by inquiry, then score “yes.”	Remove the “not applicable” response.
6. Were the characteristics of the included studies provided?	In an aggregated form such as a table, data from the original studies should be provided on the participants, interventions and outcomes. The ranges of characteristics in all the studies analyzed e.g. age, race, sex, relevant socioeconomic data, disease status, duration, severity, or other diseases should be reported. Note: Acceptable if not in table format as long as they are described as above.	As written, this question focuses on reporting and not quality.	It should be emphasized that the ranges of characteristics should be tailored to the review question.	“Not applicable” is not an appropriate response.	None.	Reword: In summary form, relevant data from the individual studies should be provided on the participants, interventions, comparators and outcomes. Note: If the summary provides the information necessary for the reader to understand the key characteristics of each study, score “yes.”	Remove the “not applicable” response.
7. Was the scientific quality of the included studies assessed and documented?	A priori methods of assessment should be provided (e.g., for effectiveness studies if the author(s) chose to include only randomized, double-blind, placebo controlled studies, or allocation concealment as inclusion criteria); for other types of studies alternative items will be relevant. Note: Can include use of a quality scoring tool or checklist (e.g., Jadad scale, risk of bias, sensitivity analysis, etc.), or a description of quality items with some kind of result for each study (“low” or “high” is fine, as long as it is clear which studies scored “low” and which scored “high”; a summary score/range for all studies is not acceptable.	The meaning of “scientific quality” is unclear. At the individual study level, an assessment of the risk of bias is likely to be more useful than consideration of quality. It is also unclear if this item refers to the individual study or to the body of evidence.	The meaning of the phrase a priori methods of assessment” is unclear. The tools used to assess risk of bias should be reliable, valid and tailored to the study design and include relevant contextual issues. Quality scoring tools are not generally recommended because they require each item to be weighted relative to other items. A sensitivity analysis is not a type of quality tool or checklist.	“Not applicable” is not an appropriate response.	Reword: Was the risk of bias assessed for each included study, taking into account important potential confounders and other sources of bias relevant to the review question?	Reword: At least two authors should assess the risk of bias using an instrument appropriate to the study design and context. A consensus process should be used to determine the final assessment. The risk of bias should be reported for each study. Quality scores should not be used; categories such as high, moderate, and low are preferred. Note: If the risk of bias of each included study was appropriately assessed and reported, score “yes.”	Remove the “not applicable” response.
8. Was the scientific quality of the included studies used appropriately in formulating conclusions?	The results of the methodological rigor and scientific quality should be considered in the analysis and the conclusions of the review, and explicitly stated in formulating recommendations. Note: Might say something such as “the results should be interpreted with caution due to poor quality of included studies.” Cannot score “yes” for question if scored “no” for question 7.	The meaning of “scientific quality” is unclear.	Systematic reviews should not contain recommendations; the difference between methodological rigor and scientific quality is unclear; and additional guidance is needed on how best to use quality assessments when formulating conclusions. The item refers only to conclusions; however the instructions refer to both analysis and conclusions. It is unclear how quality should be considered in analyses.	It is unclear how the response “not applicable” would be applied.	Reword: Was the quality of the body of evidence appropriately assessed and considered in formulating the conclusions of the review?	Reword: The review authors should have assessed the quality of the body of evidence for each important outcome across studies using GRADE or another explicit and transparent approach [37, 60, 61], and the review conclusions should reflect that assessment. Note: Score “yes” if the review authors appropriately considered the quality of the body of evidence (across studies) for each important and critical outcome in the review’s conclusions.	Remove the “not applicable” response.
9. Were the methods used to combine the findings of studies appropriate?	For the pooled results, a test should be done to ensure the studies were combinable, to assess their homogeneity (i.e., *χ* ^2^ test for homogeneity, *I* ^2^). If heterogeneity exists a random effects model should be used and/or the clinical appropriateness of combining should be taken into consideration (i.e. is it sensible to combine?). Note: Indicate “yes” if they mention or describe heterogeneity (i.e., if they explain but cannot pool because of heterogeneity/variability between interventions.	The item addresses the method for combining studies, yet the instructions relate to issues of statistical heterogeneity and imply that a meta-analysis was performed.	It is not appropriate to examine statistical heterogeneity before clinical appropriateness: the latter should always be performed first. Tests for heterogeneity do not “ensure the studies were combinable.”	None.	Reword: Were the data appropriately synthesized in a qualitative manner and if applicable, was heterogeneity assessed? If a meta-analysis was performed, was it appropriate?	Reword: Authors should provide a qualitative synthesis and explore heterogeneity if applicable. If a meta-analysis was performed, it should have been performed in an appropriate manner. Note: Score “yes” if the qualitative synthesis is appropriate, if heterogeneity was explored, and if a meta-analysis was performed, it was appropriate.	None.
10. Was the likelihood of publication bias assessed?	An assessment of publication bias should include a combination of graphical aids (e.g., funnel plot, other available tests) and/or statistical tests (e.g., Egger regression test). Note: If no test values or funnel plots included, score “no.” Score “yes” if mentions that publication bias could not be assessed because there were fewer than 10 included studies.	None.	These tests examine the issue of small study bias, not publication bias per se. Often more important than graphical and statistical tests in exploring publication bias is information that can be retrieved from study registries, and from regulatory and other agencies (e.g., gray literature).	“Not applicable” may be an appropriate response if the assessment of publication bias is inappropriate (e.g., less than 5-10 studies) or was assessed as part of the tool used to evaluate the body of the evidence (item 8).	None.	Reword: The potential for publication bias should have been considered in the review, using other information as relevant, and graphical aids and statistical tests as appropriate. The limitations of the statistical and graphical tests should be noted in the review. Note: A “yes” response can be used if the review authors explored the data and other relevant information sources for evidence of small study or publication bias. A “not applicable” response should be used if publication bias was considered as part of quality assessment of the body of evidence in item 8.	None.
11. Was the conflict of interest stated?	Potential sources of support should be clearly acknowledged in both the systematic review and the included studies. Note: To get a “yes,” must indicate source of funding or support for the systematic review AND for each of the included studies.	The phrase “conflict of interest” is unclear. This likely refers to whether there is a disclosure of conflicts, but it is unclear whether this refers to individual authors of the review and/or included studies or to the funder of the review and/or included studies.	The instructions are not congruent with the item. “Sources of support” could refer to funding for the review, financial support for the review authors, or funding of the included studies. Conflict of interest includes other interests that may interfere with the authors’ objectivity, such as personal financial interests.	“Not applicable” is not an appropriate response.	Reword: Were conflicts of interest disclosed for all of the review authors and was the funding source of the review and of each study within the review reported?	Reword: Disclosures of relevant interests should be provided for all review authors and the source of funding for the review and for each study included in the review should be reported. Note: “Yes” is indicated if disclosures of interest are provided for all review authors, the funding for the review is provided and is not likely to be a source of bias to the review’s conclusions, and the funding for all included studies is indicated (or if not reported in the individual studies then this is indicated).	Remove the “not applicable” response.
12. Proposed new Item	Not applicable	Not applicable	Not applicable	Not applicable	Were relevant subgroups considered in the review process, analysis, and conclusions?	Relevant population subgroups and characteristics should be considered in the scope and in the key questions for the review, and in searching, data extraction and analysis and in the review’s conclusions. Note: “Yes” is indicated if the main relevant subpopulations and characteristics were considered throughout the review process.	Yes, no, cannot answer

^**a**^Items, instructions, and notes listed on AMSTAR’s website (http://amstar.ca/Amstar_Checklist.php) as of June 10, 2015

## Main text

The stated objective of AMSTAR is to assess the methodological quality of systematic reviews [1] which refers to whether the authors of a study (or presumably a systematic review) did the best that they could [31]. The items of AMSTAR, however, largely address quality of reporting (e.g., items 5 and 6) [32] and risk of bias [33] (e.g., items 8 and 9) rather than the methodological quality. Several items should be amended to be consistent with the stated objective.

AMSTAR encompasses most of the key constructs that are relevant to the assessment of the methodological quality of systematic reviews; however, one critical construct is missing as noted also by other investigators [9, 34–36]: an explicit and reproducible method for assessing the quality of the body of evidence for each important outcome (i.e., the confidence in the estimates of effect [37]). We suggest revising item 8 to focus on this construct, separating it from the assessment of the quality of individual studies (item 7) (Table 1). AMSTAR also lacks an item that assesses subgroup and sensitivity analyses [9, 36]. Subgroup analyses are important to decision-makers as treatment effects may differ across populations. Similarly, sensitivity analyses specified a priori help to assess the robustness of the review’s findings [31]. Items related to subgroups and sensitivity analyses should be added (new item 12, Table 1).

Some AMSTAR items and their instructions are unclear and need to be revised (Table 1). For example, item 4 regarding the “status of publication” might refer to either the inclusion or exclusion of gray literature. The instructions suggest that gray literature should be included; however, its relevance is closely related to the review question and may not always be necessary. In AMSTAR [1], foreign language publications are considered gray literature; however, this is not consistent with commonly used definitions [38].

The response options (yes, no, cannot answer, not applicable) are problematic [9, 39–43]. For example, “cannot answer” can be difficult to interpret and distinguish from “no” when no information is provided. A common approach to quality assessment is to assume that if the authors did not report a step, then it did not happen; thus, “no” would be the appropriate response. The instructions, however, suggest that “cannot answer” should be used when the item is “relevant but not described,” which means a “no” response would rarely be used as authors seldom report explicitly that they did not do something. In addition, “not applicable” is only appropriate to two items (items 9 and 10) when these items are not possible or appropriate; all other items should always be addressed.

The guidance for scoring individual items and for obtaining a total score is unclear. In AMSTAR [1], if all criterion are met for an individual item (i.e., “yes”), it receives a score of “1” and the sum of all “yes” responses indicates the total score out of 11. Systematic reviews, however, often partially meet the item’s criteria such as listing the search databases and dates but, perhaps due to word limitations of the journal, do not provide the search strategies or keywords. To address the issue of evaluating multiple constructs within a single AMSTAR item, investigators have modified its scoring to allow points for partially fulfilled items [7, 9, 34, 35, 39]. Kung and colleagues developed R-AMSTAR [44], subdividing each item into four components with a score ranging from 11 to 44, where higher scores indicate better methodological quality. R-AMSTAR has been used by a number of investigators [5, 45–50], and a comparison to AMSTAR concluded that R-AMSTAR provided greater guidance for each item and is more reliable and useful [51].

In addition, AMSTAR provides no guidance on how to combine individual item scores from multiple assessors other than stating that consensus should be reached for each item. We have averaged AMSTAR scores across assessors to encompass each independent evaluation [52]. Other investigators have used similar approaches such as averaging scores between two assessors when discordant by one or two points and involving a third assessor when scores differed by three or more points [53, 54].

AMSTAR was deliberately developed without guidance on how to translate the total score into categorical ratings for the overall assessment of the systematic review’s quality (e.g., good, fair, poor) [1, 55]. Various thresholds have been used by investigators to define categories for quality (e.g., 0–4 vs. 0–3 for poor quality), making it difficult to compare assessments across reviews. AMSTAR was also designed under the assumption that each item is of equal weight when considering the systematic review’s overall quality [2]. Other investigators have dealt with this issue by assigning different weights to items they consider more important [53, 56–58]. For example, Jacobs and colleagues rated systematic reviews as high quality if items 3, 6, 7, and 8 were met regardless of the total score [57]. An additional problem with the current scoring method is the equivalence of “not applicable,” “no,” and “cannot answer” (all scored as zero) because an item rated as “not applicable” should not be taken into account in the total score. Clearer guidance about calculating a total score is needed along with an acknowledgement of the limitations of scoring across all items should users of AMSTAR choose to calculate a total score. We believe that obtaining a total score should be avoided as it has been shown to be problematic [59].

## Conclusion

AMSTAR is a useful tool for assessing the quality of systematic reviews; however, some modifications would improve its usability, reliability, and validity. The issues discussed in this commentary are not limited to our own experiences but are shared across many investigators who have used this tool. We have provided suggestions for improving AMSTAR; however, any revised tool needs to be empirically tested for reliability and validity, and undoubtedly, additional refinements will be needed. We look forward to further dialog on AMSTAR and to subsequent revisions and evaluations.

## Abbreviation

AMSTAR: A Measurement Tool to Assess Systematic Reviews
